# Monitoring Mechanical, Electronic, and Catalytic Trends in a Titanium Metal Organic Framework Under the Influence of Guest-Molecule Encapsulation Using Density Functional Theory

**DOI:** 10.1038/s41598-018-35117-9

**Published:** 2018-11-09

**Authors:** Hieu C. Dong, Ha L. Nguyen, Hung M. Le, Nam Thoai, Yoshiyuki Kawazoe, Duc Nguyen-Manh

**Affiliations:** 1 Center for Innovative Materials and Architectures (INOMAR), Ho Chi Minh City, 721337 Vietnam; 2 Faculty of Computer Science & Engineering, University of Technology, Vietnam National University, Ho Chi Minh City, Vietnam; 3 New Industry Creation Hatchery Center, Tohoku University, Sendai, 980-8579 Japan; 4 Culham Centre for Fusion Energy, United Kingdom Atomic Energy Authority, Culham Science Centre, Abingdon, OX14 3DB United Kingdom

## Abstract

In this study, we conduct a density functional theory investigation to study the mechanical stability of a titanium-based metal organic framework (MOF-901), which was hypothetically assumed to possess 2D characteristics. It is systematically found that the encapsulation of methanol enhances the mechanical stability of MOF-901 as the elastic tensors *C*_*ij*_ of MOF-901∙nMeOH are higher than the corresponding *C*_*ij*_ quantities reported for solvent-free MOF-901. Moreover, the 2D characteristics of MOF-901 is confirmed by verifying the negative values of *C*_33_. At the same time, the band gap of MOF-901 is observed to be solvent-dependent. In its pure form, MOF-901 possesses a direct gap (*E*_*g*_) of 2.07 eV, with the valence and conduction bands mainly constituted by electrons of 4-aminobenzoate linkers. Introducing methanol into MOF-901 causes distortion to the 4-aminobenzoate geometry, thereby induces electronic degeneracy to the conduction bands. Consequently, *E*_*g*_ is narrowed to 1.84 eV with 5.7 wt% MeOH or 1.63 eV with 11.4 wt% MeOH. Hence, it is possible to tailor the band gap of MOF-901 by controlling methanol guest, which only acquires van der Waals interaction to the framework. In addition, our theoretical prediction shows a Ti(IV) site can undergo electronic hopping to become Ti(III) under the effect of visible light (~440–443 nm). Then, Ti(III) is capable of breaking the C-Br bond in ethyl α-bromophenylacetate spontaneously, which in turn activates the polymerization of methyl methacrylate with an energy barrier of 0.30 eV.

## Introduction

Metal–Organic Frameworks, a hybrid material comprised of inorganic metal–oxo clusters and organic linking units, have been intensively studied and developed for past few decades, as proven by nearly 700,000 reported MOFs structures^1^. Possessing well-defined characteristics including high porosity, post-synthetic modification of crystal structure, structural engineering, and surface decoration, MOFs have emerged as promising candidates for key applications of global issues such as renewable energy^2^, gas storage and separation^3^, catalysis^4–6^, and drug release^7^. Among these applications, utilizing MOFs as highly tunable photocatalytic materials for catalytic transformation such as CO_2_ reduction, H_2_ production, water splitting, polymerization, and organic reactions is a key technological component of energy economics^8^. Hence, the quest for stable MOFs with promising optical properties and redox activities has led to the motivation of the MOF research community in the past decade.

For those purposes, MOFs based on tetravalent metal–oxo building units (i.e. Ti(IV), Zr(IV), and Hf(IV)) have been especially realized as promising candidates due to its high stability under harsh-working conditions^9^. Moreover, MOFs based on so-mentioned secondary building units (SBU) possess suitable band gaps for photocatalytic transformation under UV-vis irradiation which can be tailored due to the nature of MOFs structures. In order to engineer band gaps of MOFs materials, post-synthetic modification on linkers which introduces functionalities into the framework of MOFs via direct synthesis or covalent bonding, was reported by experiments as well as modeling approaches. In particular, Hendon *et al*^10^. synthesized the MIL-125-X analogues (with X = NH_2_, OH, and halogen) which exhibited a wide-tuning range of band gap from 1.1 eV to 3.6 eV. The modification of electronic structures and band gaps of MOFs were further demonstrated by Pham *et al*^11^. by employing state-of-the-art density functional theory^12–14^ (DFT) calculations to investigate the effect of functional groups to the isoreticular structures of MOF-5. Recently, Nguyen and co-workers introduced a high conjugation system of imine linking unit into MOF-901^15^, a 2D-resembled Ti-MOF constructed through imine linker and hexameric Ti–oxo cluster, to further extend the framework and reduce the band gap of the resulting material, termed MOF-902, which subsequently enhanced the photoresponsive properties compared to other competitors^16^.

By taking the advantage of molecular modeling method, band structure and electronic properties of MOFs can be fully explored and studied in details, which provides a new insight to engineer and optimize the optical response of MOFs, an imperative requirement in photocatalytic applications. In this article, we utilize molecular modeling through DFT calculations to investigate the mechanical stability of MOF-901 and present a novel approach to tailor the highest-occupied molecular orbital (HOMO)-lowest-unoccupied molecular orbital (LUMO) gap energy of this material by controlling guest molecules in the pores. We suspect that the encapsulation of solvent with the MOF structure would enhance the capability to sustain external pressure. In particular, we attempt to resolve such a hypothesis by investigating the contribution of guest molecules on the elastic tensors of the framework. In another aspect, we seek for an energetic profile of mechanism activation under visible-light irradiation of a polymerization reaction promoted by MOF-901 in the presence of ethyl α-bromophenylacetate co-initiator, which has been mentioned in the previous experimental report^15^.

## Results

### Electronic structure and mechanical stability from condensed-phase calculations

#### Electronic structure and mechanical stability of MOF-901

**Figure 1:**
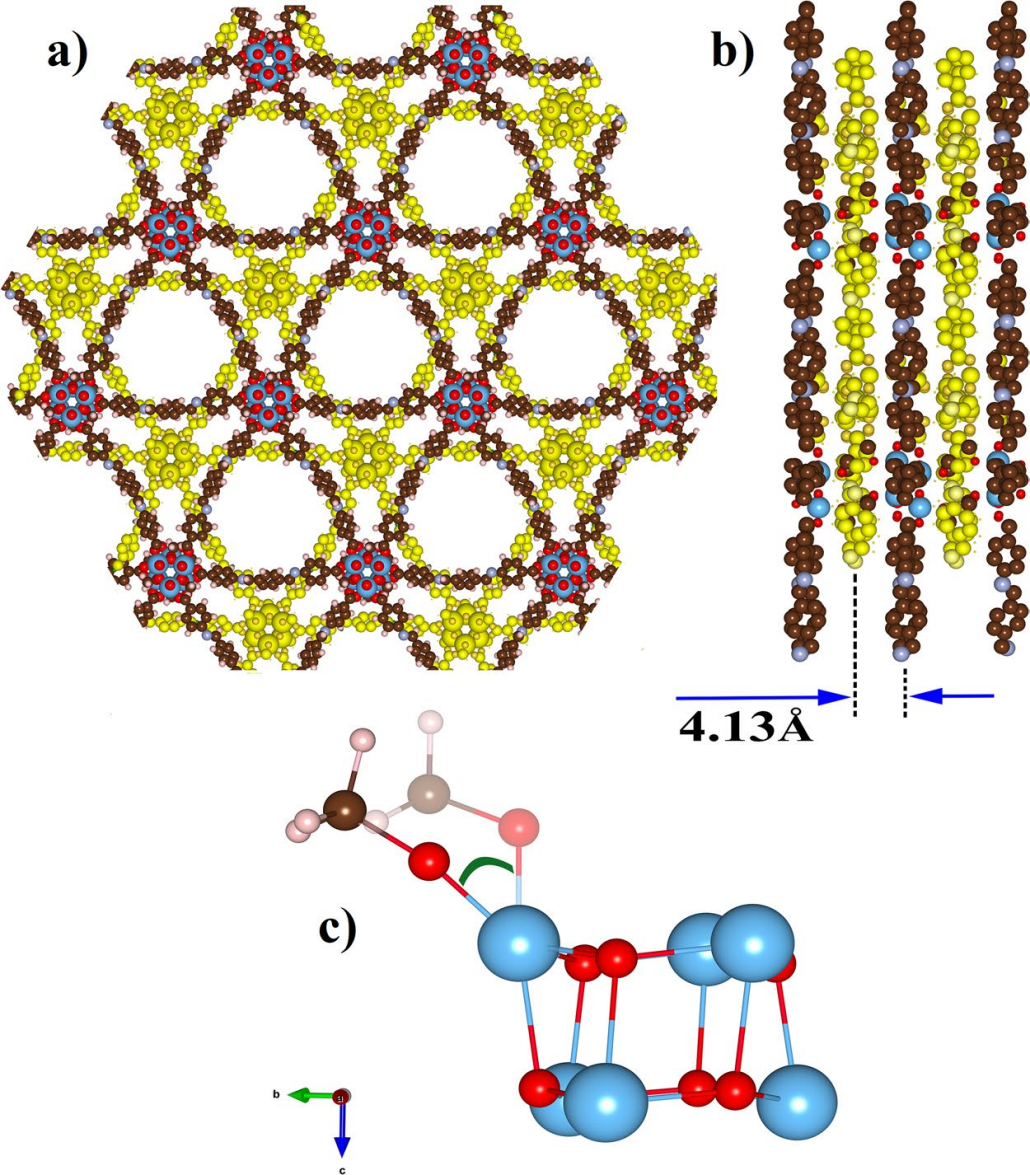
Crystal structure of MOF-901 (**a**) View from the *c* axis, (**b**) Staggered layers of MOF-901 viewed from the *a* axis showing the distance between two layers is *ca*. 4.13 Å, (**c**) An illustration of the Ti-O tilting angle (shaded color is the experimental structure). Color code: C, brown; O, red; Ti, blue; H, pink.

In a previous report, Nguyen *et al*^15^. employed power X-ray diffraction analysis to elucidate the structural information of MOF-901 (shown in Fig. 1). Upon such analysis, the unit cell parameters *a* and *c* of the hexagonal cell were reported to be 27.40 Å and 8.94 Å, respectively. In this report, we verify the structure of MOF-901 by carrying out two sets of calculations: PBE and PBE-D3 (including D3 correction terms for van der Waals interactions). After a full optimization using PBE calculations without the inclusion of empirical D3 correction, we obtain *a* to be 27.33 Å, which is in very good agreement with the experiment-reported parameter. However, the *c* lattice parameter is compressed by 7.6% (given as 8.26 Å in our calculation). In the examination of such compressing behavior, we pay most attention to the Ti-O bonds. Specifically speaking, there are two types of Ti-O bonds in the MOF-901 structure: Ti-O_(methoxy)_ and Ti-O_(carboxylate)_. The Ti-O_(carboxylate)_ bond is established within the [Ti_6_O_6_(O-CH_3_)_6_(4-aminobenzoate)_6_] core cluster as first synthesized by Hong and Chun^17^, while the Ti-O_(methoxy)_ bond links Ti to a methoxy group. The available experimental data also revealed that the two types of Ti-O bonds fell in the range of 1.98–2.00 Å. From our DFT optimization, the Ti-O_(carboxylate)_ bond is extended to 2.12 Å, while the Ti-O_(methoxy)_ bond is compressed in comparison with the experimental values. The compression of Ti-O_(methoxy)_ bonds is associated with the compression of the *c* lattice parameter. In addition, we also observe that both Ti-O_(methoxy)_ and Ti-O_(carboxylate)_ bonds are tilted (Fig. 1), while in experiment, those two bonds are shown almost parallel to the z axis. Introducing the D3 correction for long-range van der Waals interactions, in principle, would enhance long-range binding energetics. With D3 correction accounted, we find the *a* and *c* lattice parameters to be 27.27 Å and 8.55 Å, respectively. It should be noted that the *c* axis seems to suffer less from lattice narrowing of the PBE-D3 optimization. In this case, the Ti-O_(methoxy)_ and Ti-O_(carboxylate)_ bonds are 2.06 Å and 1.90 Å, respectively, slightly smaller than the previous PBE-optimized values. As a result, we can verify that the compressed *c* lattice is caused by Ti-O tilting behavior. In addition, it should be noted that in our calculations, the long-range van der Waals interactions cannot be perfectly described by the empirical D3 correction. For validation of pseudopotentials, we perform another optimization with a consideration of semicore electron inclusion. At convergence, the *c* lattice parameter is more compressed in comparison with the previous PBE results. For convenience, we list the resultant *a*, *c* lattice parameters and unit-cell volume in each calculation in Table 1.

**Table 1:** Lattice parameters and volumes of solvent-free MOF-901, MOF-901 with 5.7 wt% MeOH, and MOF-901 with 11.4 wt% MeOH.

Structure	Calculation method	a (Å)	c (Å)	Volume (Å^3^)	Ti-O_(methoxy)_ (Å)	Ti-O_(carboxylate)_ (Å)
MOF-901	Experiment	27.40	8.94	5,813	2.00	1.95
	PBE	27.33	8.26	5,343	1.79	2.12
	PBE + D3	27.27	8.55	5,506	1.90	2.06
MOF-901 with 5.7 wt% MeOH	Experiment	27.30	8.38	5,409	1.99	1.98
	PBE	27.44	8.05	5,249	1.79	2.12
	PBE + D3	27.27	7.84	5,049	1.78	2.11
MOF-901 with 11.4 wt% MeOH	Experiment	27.36	8.04	5,212	1.99	1.98
	PBE	27.40	8.13	5,286	1.79	2.10
	PBE + D3	27.29	8.20	5,289	1.78	2.10

**Figure 2:**
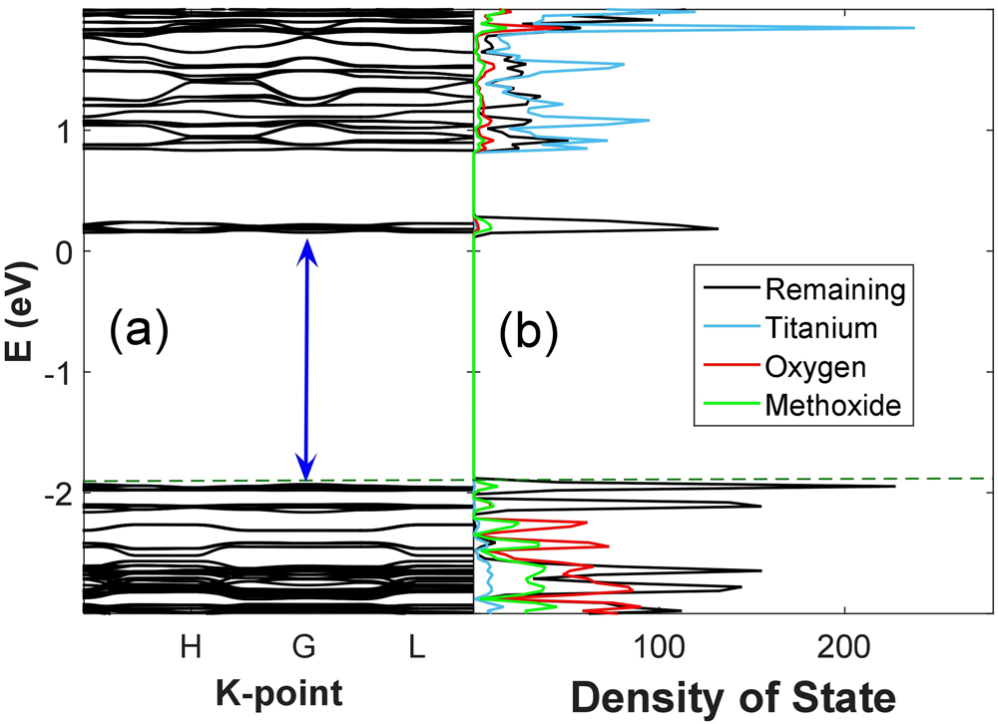
(**a**) Band structure of hexagonal MOF-901, (**b**) PDOS of Ti, O(carboxylate), methoxy, and 4-aminobenzoate given by PBE calculations.

From electronic structure analysis of PBE calculations, MOF-901 is found to possess a direct HOMO-LUMO gap of 2.07 eV at the Γ point as shown in the band structure in Fig. 2a. Compared with the optical band gap (2.65 eV) measured by UV-vis diffuse reflectance spectroscopy^15^, our calculated *E*_*g*_ herein is underestimated by 22%. To our knowledge, the resultant percent error is acceptable, considering the fact that PBE calculations always underestimate *E*_*g*_ as a result of poor electron-hole interaction description in the exchange-correlation functional^18^. In a typical case of band-gap underestimation, the problematic circumstance arises severely when the 3d electrons of metal directly constitute either the HOMO or LUMO levels. Here, our situation is quite different. In this particular case of MOF-901, both HOMO and LUMO are, however, localized on the organic linkers. Thus, the band gap prediction herein does not suffer much from underestimation. When analyzing the HOMO-LUMO gap of PBE-D3 calculations, a direct gap of 2.03 eV is observed, which is slightly lower than the quantity given by conventional PBE calculations.

Subsequently, we examine the contribution of molecular orbital to the nature of HOMO-LUMO gap opening for MOF-901 by analyzing the partial density of states (PDOS) of four different groups: (1) Ti, (2) O_(carboxylate)_, (3) methoxy, and (4) remaining 4-aminobenzoate (Fig. 2b). Even though Ti is considered as an important recipe to construct the building block for hexagonal cell formation, it is surprisingly revealed that the contribution of Ti to the overall HOMO-LUMO gap is insignificant. The cationic charge of Ti is revealed as + 1.26 without D3 correction, or + 1.25 with D3 correction. The covalent group, i.e. 4-aminobenzoate, plays a very important role instead. As we can see from the PDOS plot (Fig. 2b), the eigenstates of HOMO and LUMO are mainly constituted by the hybridized orbitals of 4-aminobenzoate, whereas the 2*p* orbitals of O_(carboxylate)_ atoms dedicate a smaller contribution.

Besides the theoretical investigation of electronic structure, we also validate the mechanical stability of MOF-901 by evaluating the *C*_*ij*_ elastic tensors^19^ resulted from our DFT calculations. To evaluate the unit-cell elastic response, we employ a central finite difference scheme with two ionic (or lattice) displacements (one positive and one negative) for computational feasibility. For a large structure of 336 atoms within a hexagonal unit cell, and thanks to symmetry consideration, we only need to evaluate 168 degrees of freedom for Hessian evaluation in total. The elastic tensors with consideration of rigid ions and with ionic response inclusion are reported in Table 2. It should be noted, however, that we only reported those elastic tensors given by conventional PBE calculations. The PBE-D3 calculations are not chosen in this case because of empirical fitting in the D3 parameterization, which certainly alters the MOF structure from its actual ground state given by PBE calculations.

**Tablee 2:** Elastic tensor moduli (kBar) of pure MOF-901 arising from PBE calculations.

Elastic mode	*C*_*11*_	*C*_*12*_	*C*_*13*_	*C*_*33*_	*C*_*44*_	*C*_*66*_
Symmetrized elastic modulus (without ionic contribution)	2504	526	405	921	526	988
Total elastic modulus (with ionic contribution)	487	150	33	−14	−1	168

For a particular hexagonal system, the mechanical stability can be certified if all four following conditions hold^20,21^

1 $${C}_{{11}} > |{C}_{{12}}|$$

2 $$2{{C}_{{13}}}^{{2}} < {C}_{{33}}({C}_{{11}}+{C}_{{12}})$$

3 $${C}_{{44}} > 0$$

4 $${C}_{{66}}=({C}_{{11}}-{C}_{{12}})/2$$

In the original formalism to calculate elastic constants using the stress-strain relationship^22^, only lattice distortions (with rigid ions) are considered, and the listed *C*_*ij*_ values considering rigid ions in Table 2 provide complete satisfaction to the above inequalities. In other words, MOF-901 can be confirmed to be stable by the elastic tensors derived from lattice distortions. On the other hand, if the approximate harmonic ionic response is taken into account, *C*_*33*_ and *C*_*44*_ become negative after adding the largely negative terms from ionic response, leading to the unsatisfaction of inequalities (2) and (3). The very small value of *C*_*44*_ (−1 kBar) may be a result of calculation deviation from lattice deformation and ionic perturbation in a very large unit cell. As we acknowledge the compression of the *c* lattice parameter in the earlier part of this discussion, obtaining a negative value of *C*_*33*_ with ionic response inclusion is not surprising. Upon the compression in the z direction, the two types of Ti-O bonds listed above seem to suffer from bending, not from bond compression. More interestingly, the negative value of *C*_*33*_ truly indicates the 2D-characteristics of MOF-901. It should be noted that the z-oriented connection between layers of MOF-901 is established by an alternative zigzag bonding scheme through 4-aminobenzoate, as illustrated in Fig. 1b. Overall, upon consideration of elastic tensors given by unit-cell compression, we conclude that MOF-901 is mechanically stable with 2D characteristics.

#### Methanol encapsulation with MOF-901: change in electronic structure and effect on mechanical stability

As a solvent in the chemical synthesis, methanol is naturally encapsulated within the pores of MOF-901. Here, we validate different concentrations of methanol adsorbed inside the network. In general, the adsorption energy of multiple methanol molecules inside MOF-901 can be calculated using the following equation:

5 $${E}_{adsorption}={E}_{n\times methanol@MOF-{901}}-{E}_{MOF-{901}}-{\rm{n}}{E}_{methanol}$$

where *E*_*MOF-901*_, *E*_*methanol*_, and *E*_*n×methanol@MOF-901*_ are the total energies of MOF-901, an isolated methanol molecule, and MOF-901 with *n* methanol molecules, respectively. A negative value of *E*_*adsorption*_ indicates attraction between methanol and MOF-901, while a positive value supposingly indicates repulsion.

There are two levels of methanol adsorption concentration inside MOF-901 as reported in a previous experiment^15^. In the first case, 6 methanol molecules are encapsulated within a MOF-901 primitive cell (5.7 wt%). In the second case, the adsorption concentration is doubled (12 methanol molecules, 11.4 wt%). The structural configurations obtained from power X-ray diffraction reveals that the hexagonal unit cell is nicely retained in both adsorption cases. Those configurations are used in our optimizations.

Subsequently, we perform optimizations for those two structures using the same calculation method. In both cases, methanol tends to establish van der Waals interaction with the six-membered 4-aminobenzoate moiety. Interestingly, this long-range interacting behavior is correlated with an impact on electronic structure that we will discuss later on. To analyze the favorability of settlement, the adsorption energy is subsequently calculated. For PBE calculations without D3 corrections, 6 methanol molecules are shown to expose attraction to the framework (−0.23 eV/molecule). PBE-D3 calculations reveal that 6 methanol molecules find nice dwelling in MOF-901 pores with an improved adsorption energy of −0.42 eV/molecule, as shown in Table 3.

**Table 3:** Adsorption energy of various MeOH concentration in MOF-901 (eV/molecule).

Calculation method	PBE	PBE + D3
5.7 wt% MeOH	−0.23	−0.42
11.4 wt% MeOH	−0.40	−0.52
13.3 wt% MeOH	−0.33	−0.61

**Figure 3:**
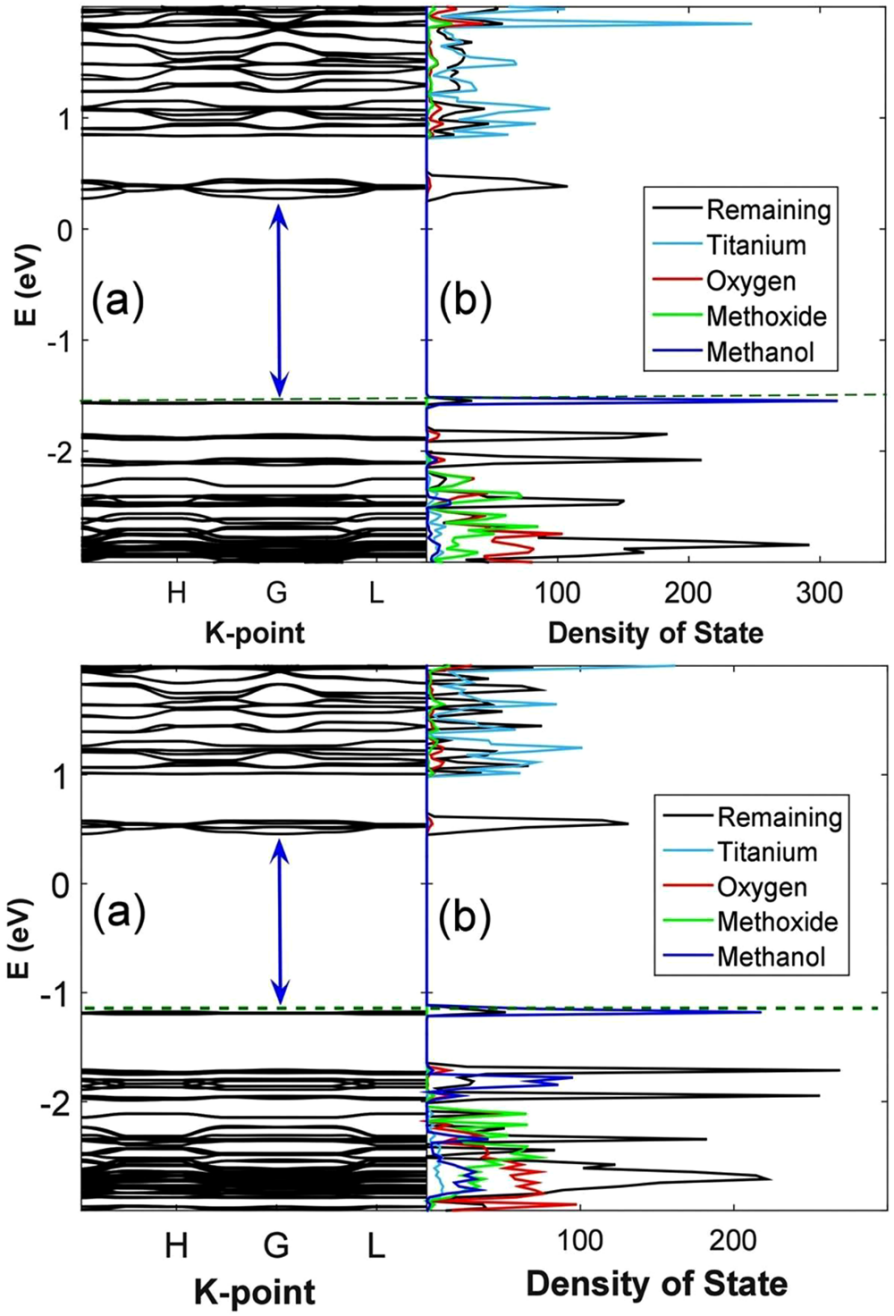
(**a**) Band structure of MOF-901 containing 6 guest methanol molecules (5.7 wt%) and PDOS of Ti, O(carboxylate), methoxy, methanol, and 4-aminobenzoate (remaining), (**b**) band structure of MOF-901 containing 12 guest methanol molecules (11.4 wt%) and PDOS of Ti, O(carboxylate), methoxy, methanol, and 4-aminobenzoate (remaining) given by PBE calculations.

The *c* lattice parameter is shortened, while *a* almost remains unchanged. Looking at the two-dimensional characteristics in Fig. 1b, it is reasonable that MeOH establishes long-range interaction with 4-aminobenzoate linkers along the z direction, and acts as a bridge to connect two parallel panels (layers). Hence, the *c* lattice parameter is shortened to 7.84 Å. The magnitude of binding energy given by PBE-D3 calculations unsurprisingly indicates weak van der Waals interactions. At this stage, we are curious to learn how this concentration of solvent would affect the electronic property of MOF-901. According to band structure and PDOS examination, *E*_*g*_ is now narrowed to 1.84 eV as predicted by PBE calculations as shown in Fig. 3a.

Recall that in pure MOF-901, both HOMO and LUMO are mainly constituted by the hybridized orbitals of 4-aminobenzoate. In the MOF-methanol interacting model, methanol establishes long-range interaction with the phenyl ring and distorts 4-aminobenzoate away from the original equilibrium position, thereby causes an incidental alternation on the electronic structure. Encountering structural distortion from the equilibrium position, the LUMO band is broadened around the region of 0.3–0.5 eV. Interestingly, there is also a split in the LUMO band as shown in the PDOS plot (Fig. 3b), indicating that there is an energetic degeneracy in the virtual eigenstates as a result of structural deformation of 4-aminobenzoate. Moreover, an in-gap state appears near the Fermi level, which is constituted by almost-equal contributions of guest methanol and 4-aminobenzoate. At this point, we can point out two factors causing *E*_*g*_ narrowing. First, guest molecules themselves contribute an intermediate eigenstate in the valence region. Second, the spatial occupancy of MeOH induces distortion to 4-aminobenzoate, and eventually broadens LUMO. It is also interesting to notice that the Fermi level is shifted to a higher energy level (1.5 eV) under the presence of methanol.

The most important goal in this study is to verify elevation of mechanical stability under the presence of methanol inside the MOF-901 network. This can be achieved by conducting elastic moduli calculations. The motivation for elasticity verification of MOF-solvent systems relies on the fact that a MOF product obtained from an experiment usually needs to undergo a stability test after product activation. Therefore, we suspect that the calculated elastic tensors *C*_*ij*_ would indicate MOF-solvent to be more mechanically-stable than the pure MOF itself (activated sample). Upon analyzing elastic tensors resulted from lattice distortion, we observe that the *C*_*ij*_ values for MOF-901 with 5.7 wt% methanol increase from 3% to 17% (see Tables 2 and 4). When ionic relaxation is included, we again observe that *C*_*33*_ is negative (−155 kBar), while *C*_*13*_ is largely reduced. This is an implication of 2D characteristics. More importantly, the elastic tensor moduli show that MOF-901 with 5.7 wt% methanol is more mechanically stable than pure MOF-901.

**Table 4:** Elastic tensor moduli (kBar) of MOF-901 encapsulating guest methanol molecules arising from PBE calculations.

		*C*_*11*_	*C*_*12*_	*C*_*13*_	*C*_*33*_	*C*_*44*_	*C*_*66*_
Symmetrized elastic modulus (without ionic contribution)	MOF-901 with 5.7 wt% MeOH	2604	542	469	1031	617	1031
	MOF-901 with 11.4 wt% MeOH	2608	543	497	1152	665	1032
	MOF-901 with 13.3 wt% MeOH	2971	600	484	1244	650	1158
Total elastic modulus (with ionic contribution)	MOF-901 with 5.7 wt% MeOH	526	139	−119	−155	−70	193
	MOF-901 with 11.4 wt% MeOH	535	272	−47	−157	−25	131
	MOF-901 with 13.3 wt% MeOH	313	15	−186	−203	−46	135

Under the presence of 12 methanol molecules, the *c* lattice parameter is even less shortened than in the case of 6-methanol adsorption as listed in Table 1. In this case, while PBE indicates attraction with an adsorption energy of −0.40 eV/molecule, PBE-D3 gives an adsorption energy of −0.52 eV/molecule. Examining the electronic structure of MOF-901 with 11.4 wt% methanol in Fig. 3b, *E*_*g*_ is observed to reduce to 1.63 eV. From the PDOS plot, we observe contribution of both methanol and 4-aminobenzoate to the formation of HOMO, while the lowest-unoccupied state is constructed by 4-aminobenzoate like the previous case. It should be noticed, however, the contribution of methanol to the in-gap state herein is much more dominant than that in the 6-methanol case. In terms of mechanical stability, we observe that the resultant elastic moduli for MOF-901 with 11.4 wt% methanol are further stabilized in comparison with the previous 5.7 wt% methanol case. More specifically, *C*_*ij*_ values given by lattice distortion increase by 4–26%. In general, *C*_*ij*_ given by lattice distortion are larger than those values obtained for pure MOF-901 and MOF-901 with 5.7 wt% methanol. As ionic response is taken into account, it should be noticed that *C*_*33*_ is -157 kBar, which again implies the 2D characteristics the framework. Equation (4) is satisfied, which implies that the MOF structure containing methanol still possesses hexagonal symmetry.

**Figure 4:**
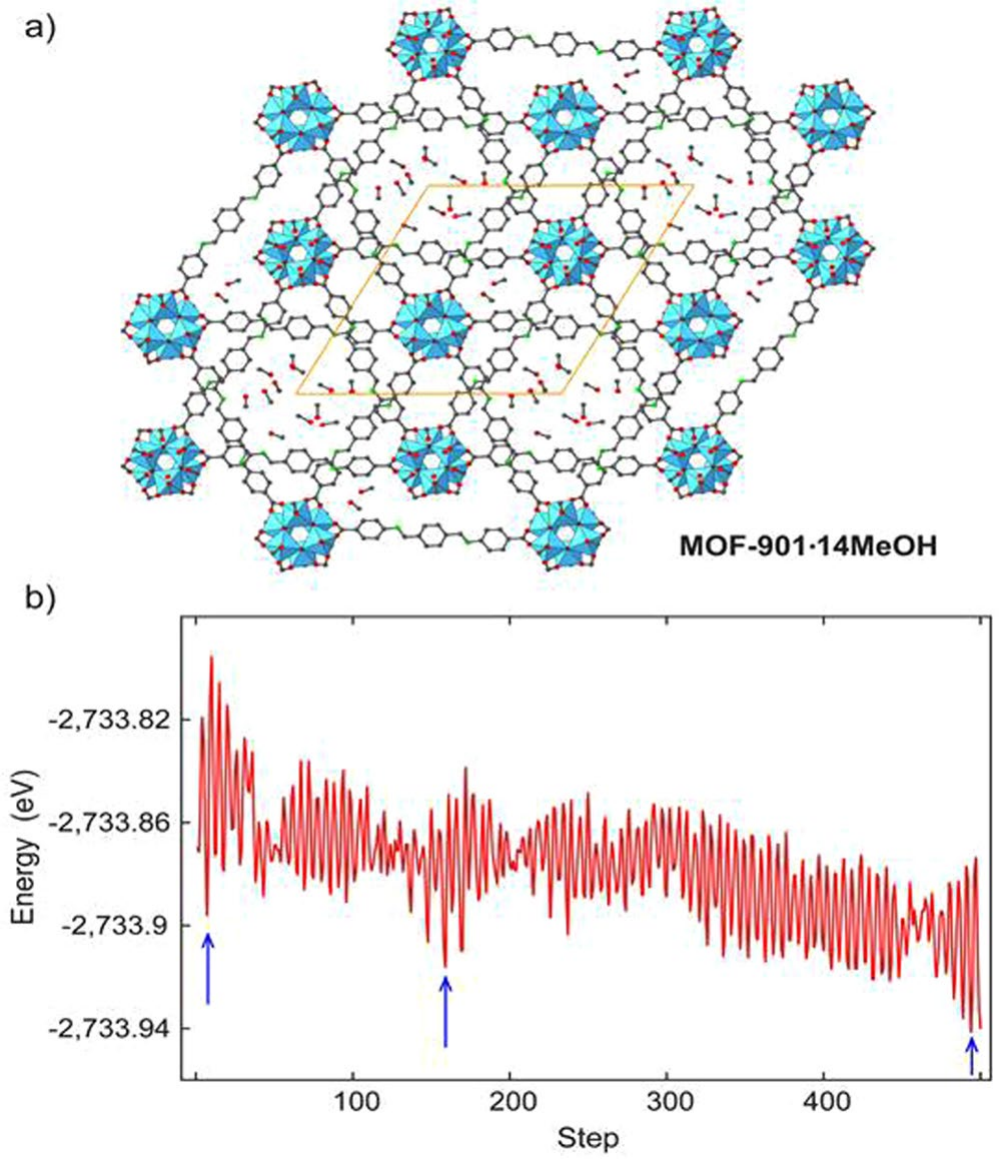
(**a**) MOF-901 with a load of 14 methanol molecules and (**a**) time-energy diagram. Three blue arrows in the energy diagram indicate three chosen configurations where we believe the local minima reside. Color code: C, black; O, red; N, green; Ti, blue polyhedra. H atoms are omitted for clarity.

In the third case, we hypothetically push up the concentration of solvent to 13.5 wt% (14 methanol molecules). In this hypothesized structure, we assume that methanol can further find settlement in the small pore of MOF-901, which might not be observed in experiment spectrometers. Recall that in the previous adsorption cases, it is demonstrated in experiment that methanol only adsorbs in the large pore. In this case, 14 methanol molecules are placed in both large and small pores randomly. Due to complexity of methanol arrangement, we carry out a molecular dynamic simulation using the PBE functional with a fixed step size of 0.5 fs and fixed unit cell parameters at 300 K. This dynamic process enables us to search for local minimum configurations. We select three configurations of methanol arrangements (blue arrows in the time-energy diagram in Fig. 4) at three different time frames to get variant arrangement of the methanol molecules. The total time period for molecular dynamic simulation is 0.25 ps. Then, the chosen structures are optimized with variant unit cell, and the adsorption energy is calculated as -0.33 eV/molecule without D3 correction or -0.61 eV/molecule with D3 correction. In this case, both PBE and PBE-D3 calculations show that 14-MeOH-molecule encapsulation is even more attractive to the network of MOF-901, which means that MeOH also prefers to occupy small pores. The next goal is to validate mechanical stability of MOF-901 with 14 methanol molecules encapsulation.

The calculated elastic tensors are reported in Table 4. The *C*_*ij*_ values given by unit-cell differentiating increases at least 14% in comparison with the value of pure MOF-901. More specifically, *C*_*33*_ (1244 kbar) increases tremendously by 35% in comparison with that of pure MOF-901 (921 kbar). Of course, these values survive the tests from inequalities (1)–(3). As we consider ionic relaxation contribution into total elastic tensors, *C*_*33*_ and *C*_*13*_ are more negative than those reported for the previous pure MOF-901 case.

At this point, our hypothesis has been resolved. The elastic tensor data clearly indicates that solvent (methanol) actually fills the vacuum porosity in MOF-901, thus enhance the mechanical stability of the structure. Elastic tensor data in Table 4 also implies the 2D characteristics of MOF-901. In terms of electronic structure, the appearance of guest molecule (MeOH) in MOF-901 pores causes geometry distortion to phenyl rings, thereby narrows the HOMO-LUMO gap.

#### Dehalogenation of ethyl α-bromophenylacetate at the electronically excited Ti site of [Ti_6_O_6_(O-CH_3_)_6_(4-aminobenzoate)_6_]

**Figure 5:**
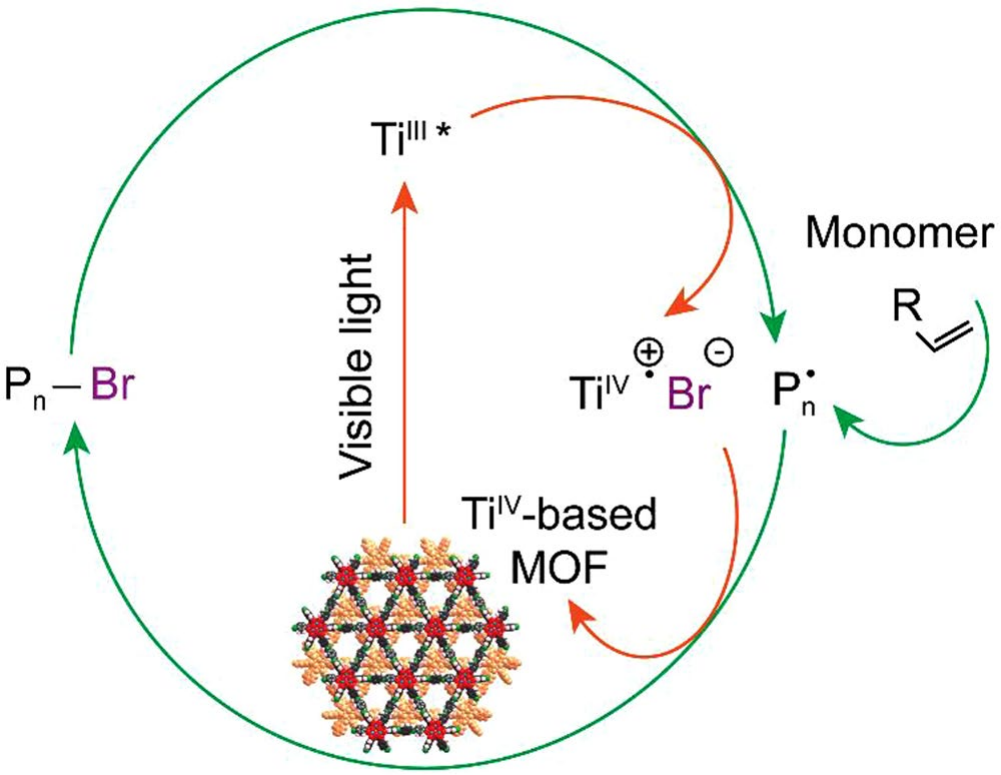
Mechanism for UV-vis irradiation of a polymerization reaction promoted by MOF-901 in the presence of ethyl α-bromophenylacetate as a co-initiator.

Experimentally^15^, the photocatalytic activity of MOF-901 was demonstrated in the polymerization of methyl methacrylate with ethyl α-bromophenylacetate as a co-initiator as shown in Fig. 5. With the motivation from the experiment, we theoretically demonstrate in this section that Br can be removed from ethyl α-bromophenylacetate (dehalogenation) under the influence of active Ti(III) sites. According to the electronic DOS (Fig. 2), the 3d electrons of Ti contribute partially to build up the HOMO-2 and LUMO+1 level, which corresponds to a hopping energy of 2.93 eV (423 nm). The oxidation state hopping energy was investigated in various theoretical and experimental works. For example, magnetic exchange-coupling constant was investigated for di- and trinuclear transition metal complexes by employing various ab initio levels of theory. For dicobalt complexes, the spin-orbit coupling effect had to be taken into account to obtain qualitative results^23^. Investigating the mechanism of SBU in a chromium-based MOF, Cantu *et al*^24^. suggested the highest barrier of ~35 kcal/mol for the formation of a dimetal linker and high-low spin hopping. The flip of Ti oxidation state from IV to III has been shown to occur under the influence of visible light in the literature. In a previous experiment, Dan-Hardi and co-workers^25^ synthesized a MOF structure involving titanium-oxo-hydroxo and dicarboxylate linkers (namely MIL-125). Interestingly, an optically-activated electronic hopping was observed by the evidence of electron paramagnetic resonance spectroscopy, and the oxidation state of Ti was reported to switch from IV to III^26^. Under the photo-activation of UV-A light, the Ti(IV) sites in COK-69 was reported to successfully convert to Ti(III)^27^. MIL-101, on the other hand, was constructed solely by Ti(III), and possessed a capability to capture O_2_ at the Ti site (then, the Ti oxidation state became IV after bonding with O_2_)^28^. For our case, it is of importance to verify the possibility of switching oxidation state of Ti to further study the mechanism of polymerization reaction under visible light irradiation catalyzed by MOF-901. An isolated model of Ti_6_O_6_(OCH_3_)_6_(4-aminobenzoate)_6_ is considered, which consists of 138 atoms and possesses *C*_*3v*_ symmetry.

**Figure 6:**
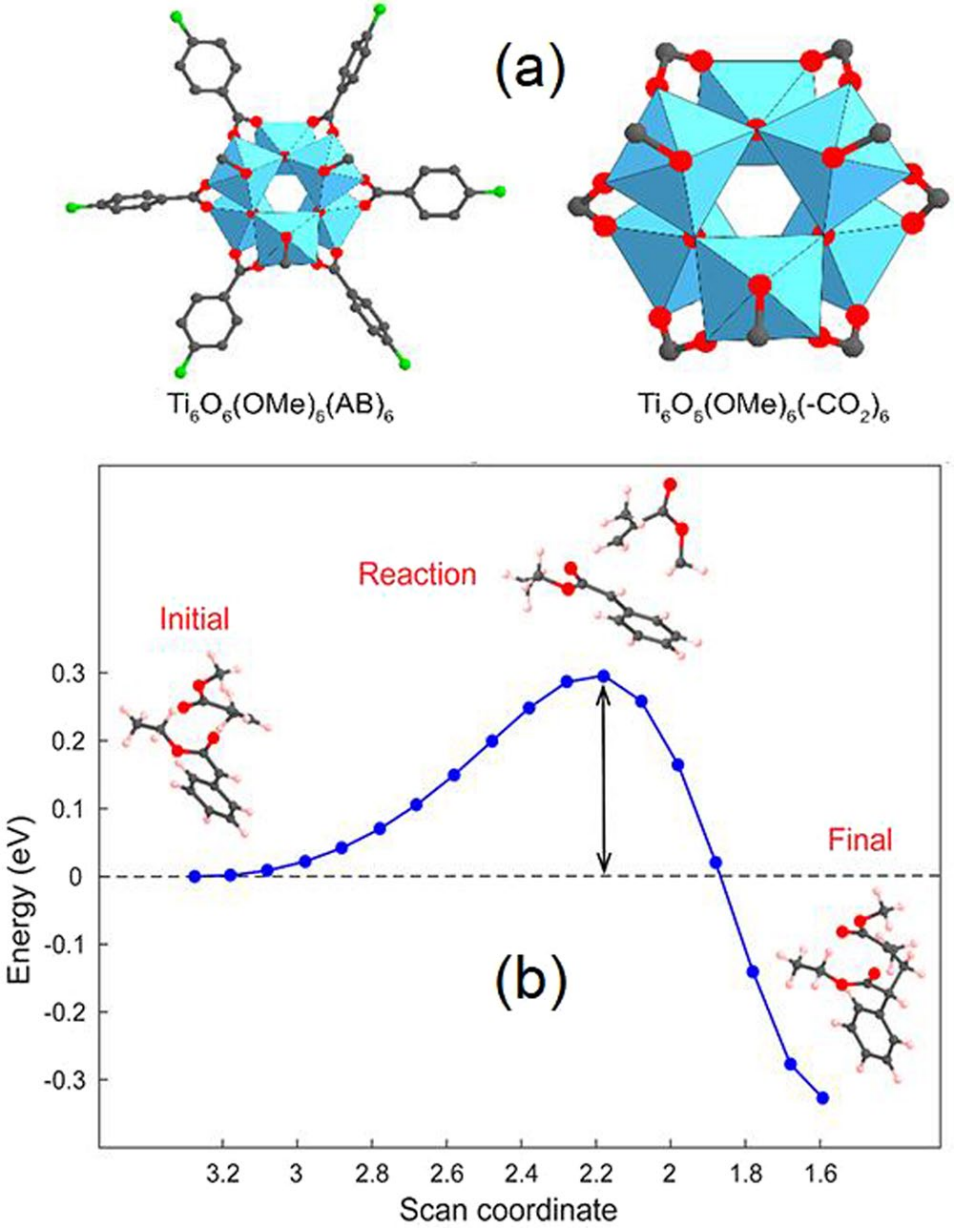
(**a**) Singlet-state structure of [Ti_6_O_6_(OMe)_6_(AB)_6_] cluster (AB = 4-aminobenzoate) and the simplified model in which AB groups are replaced by formate functionalities (HCO_2_) to save computational effort. Color code: C, black; O, red; N, green; Ti, blue polyhedra. H atoms are omitted for clarity. (**b**) Energy profile of the polymerization of methyl methacrylate under the activation of radical phenylacetate showing a barrier of 0.30 eV.

In the singlet ground state of Ti_6_O_6_(OCH_3_)_6_(4-aminobenzoate)_6_, when Ti switches the oxidation state from IV to III, the methoxy (•OCH_3_) radical group should be released as a consequence. Moreover, there is another lone-pair electron at the Ti site. Therefore, the overall multiplicity should be triplet. Indeed, achieving self-consistency in total energy calculation for this triplet state is hardly done; therefore, for such a highly complex molecular system, we perform two doublet calculations instead: one for •OCH_3_ and another for the remaining cluster. The sum of total energies of two doublet configurations is 4.19 eV higher than the singlet energy of Ti_6_O_6_(O-CH_3_)_6_(4-aminobenzoate)_6_ according to PBE/6–31 G calculations or 3.97 eV lower according to PBE/*gen* calculations. These predicted energy differences correspond to UV-A wavelengths of 296 nm or 312 nm, respectively. To get a more realistic picture of the singlet-triplet hopping energy, we consider a simplified cluster, in which the 4-aminobenzoate moiety is omitted and replaced by a dummy H atom, while all -OCH_3_ groups still remain in the complex (66 atoms in total). It is still very challenging to achieve self-consistency for triplet-state calculations, and we have to employ a quadratically-convergent method for solving Hartree-Fock SCF^29^. For this model, upon using PBE/6–31 G, the singlet-triplet hopping energy is 2.82 eV (440 nm, in the visible region), while PBE/gen suggests a hopping energy of 2.80 eV (443 nm). Those two hopping energies indicate good correspondence with the earlier plane-wave calculations and more importantly, possess good agreement with the actual condition for polymerization, where the reaction was catalyzed under visible light^15^. At this stage, we look forward to validating the removal of Br from ethyl α-bromophenylacetate at the Ti active site (without •OCH_3_) of the simplified model. In the reaction, Br approaches Ti(III) from a far distance. Ti(III) then captures Br to establish a Ti-Br ionic bond; then, the C-Br bond is broken as a consequence. To achieve a transition state for this proposed mechanism, we first optimize an initial state, where ethyl α-bromophenylacetate is set to interact with the core cluster from a far distance (to be specific, more than 6 Å), and a final state, where Br is successfully captured by Ti and radical ethyl phenylacetate is left behind. While the final state can be successfully optimized, it is interesting to observe from both PBE/6–31 G and PBE/*gen* calculations that an initial state cannot be found in both sets of calculations; in fact, the initial structure is dragged to converge toward the final state. Hence, dehalogenation under the activation of Ti(III) is a barrierless and thermodynamically spontaneous process because Ti(III) is very active. Thereafter, radical ethyl phenylacetate is produced. We then carry out the key step of polymerization using the gas-phase calculation by allowing the radical C site in ethyl phenylacetate to approach the double bond in methyl methacrylate, as shown in Fig. 6b. In this optimization, the C(radical)-C(double bond) distance is narrowed by a step size of 0.1 Å, while other coordinates are relaxed. As the transition state is located, the radical C atom from ethyl phenylacetate approaches the *sp*^2^ C atom in methyl methacrylate with a distance of 2.18 Å, and the potential barrier is 0.30 eV. The Gibbs free energy of this polymerization is 0.37 eV, thus the reaction rate constant at room temperature can be estimated as 3.43·10^6^ s^−1^ using the Eyring equation^30^. We believe such a reaction barrier is low enough to be activated in ambient condition. Nevertheless, such reaction free energy and rate constant are estimated in the gas phase. Within the context of this report, we do not carry out a demonstration of polymerization in the condensed phase within the pores of MOF-901; still, we believe that the reaction rate would be slower.

## Conclusion

In this study, we carry out a theoretical investigation of MOF-901 to clarify the difference in mechanical stability of solvent-free and solvent-interacting systems. Structural optimizations predict the *a* lattice parameter to be in good accordance with the experiment, while the *c* lattice is compressed due to the tilting behavior of −OCH_3_. The adsorption of methanol is proved to stabilize the framework of MOF-901. The analysis of elastic tensors *C*_*ij*_ for solvent-free MOF-901 and MOF-901 with solvent indicates that both systems are mechanically stable and possess 2D characteristics. Moreover, it also unveils the role of solvent in stabilizing the porous material. Upon introducing methanol into MOF-901, all elastic moduli *C*_*ij*_ derived from a stress-strain relationship increase. This is an indication that the framework has attained better structural stability. We then examine the band structure of MOF-901 given by PBE calculations, and observe a direct HOMO-LUMO gap of 2.07 eV at the Γ point. From the PDOS analysis, both HOMO and LUMO are mainly constituted by the hybdrized orbitals of 4-aminobenzoate groups, while the contributions from Ti, O_(carboxylate)_, and methoxy are insignificant. The 5.7 wt% encapsulation of methanol within MOF-901 pores (6 methanol molecules) narrows the energy gap to 1.84 eV (decreased by ~12%). By further increasing methanol concentration to 11.4 wt% (12 methanol molecules), the HOMO-LUMO gap suffers more narrowing (1.63 eV) as a result of distorting the equilibrium geometry of 4-aminobenzoate linkers. It should be noted that those two methanol-incorporating structures are obtained from a previous experimental data. Guest methanol itself contributes an in-gap state, which is also responsible for *E*_*g*_ narrowing. This finding opens up a new direction to MOF energy gap tailoring by control solvent concentration.

Finally, the role of Ti(III) in dehalogenation of ethyl α-bromophenylacetate is verified. Our localized atomic-orbital-basis calculations suggest that a Ti(IV) site can undergo electronic hopping to become Ti(III) under the effect of visible light (~440–443 nm). Subsequently, Ti(III) is capable of grabbing Br from a far distance and break the C-Br bond in a spontaneous process without a reaction barrier. Finally, the polymerization of methyl methacrylate can be activated by radical ethyl phenylacetate with a low reaction barrier of 0.30 eV.

## Methods

### Condensed-phase calculations

Plane-wave DFT calculations are performed for the investigation of MOF-901. With a hexagonal structure, MOF-901 consists of 336 atoms within one primary unit cell (12 Ti, 12 N, 144 C, 120 H, and 48 O atoms) as shown in Fig. 1. The total charge of the system of interest vanishes because all atoms are chemically guaranteed. Electronic structure calculations for periodic MOF-901 are executed using the well-established Vienna Ab Initio Simulation Package (VASP)^31–34^. To be specific, DFT calculations are performed by employing the Perdew-Burke-Ernzerhof^35,36^ (PBE) parameterization for exchange-correlation description within generalized-gradient approximations (GGA). The electronic wave-function is constituted using the well-developed projector-augmented wave (PAW) method implemented within VASP^37,38^. The cut-off energy for plane-wave expansions is tested at various values from 300 eV to 425 eV, and we observe that total energy is lowest at 400 eV. Therefore, we choose the cut-off energy of 400 eV, which is most suitable and affords computational feasibility for such a large periodic system. The convergence threshold in total energy self-consistency is 10^−5^ eV. Each optimization process has two steps because of very large and porous structure. In the first step, the unit cell is fixed to retain structural symmetry and ionic position is optimized until total energy difference between self-consistent-field (SCF) loops falls within an allowable limit of 10^−3^ eV. Subsequently, the unit cell and ionic positions are relaxed simultaneously. In the second step, a convergence criterion of 10^−3^ eVÅ^−1^ is applied to gradient convergence. For illustration of structures, crystallographic pictures are made with the Visualization for Electronic and Structural Analysis software^39^.

We also study methanol encapsulation inside MOF-901 in a later section. To survey the geometric arrangement of multiple methanol molecules, we first introduce 14 CH_3_OH molecules into MOF-901 randomly; then, a molecular dynamic trajectory is employed to shuffle CH_3_OH. The constant temperature of 300 K is acquired by utilizing the Andersen thermostat technique^40,41^, and the total time of this simulation is 0.25 ps (500 molecular dynamic steps).

### Localized atomic-orbital-basis calculations

The localized atomic-orbital-basis calculations are executed using unrestricted PBE calculations implemented in the Gaussian 16 suite of program^42^. Two independent calculation sets are performed for validation purposes. In the first set, we employ the simple split-valence Pople basis set of 6-31 G^43,44^. In the second calculation set, an effective core potential^45^ with the LANL2dz basis set^46–48^ (for convenience, we denote the combination of basis sets and effective core potential as *gen*) is employed. The long-range van der Waals interaction is accounted in those optimizations by activating the empirical Grimme D3 correction terms^49^.
